# Supplementary material for: Multi‐dataset identification of innovative feature genes and molecular mechanisms in keratoconus
Source: J Cell Mol Med. 2024 Sep 19;28(18):e70079. doi: 10.1111/jcmm.70079 (PMC11412914; doi:10.1111/jcmm.70079)
Supplement: Supplementary file 2 — Material S2. [file JCMM-28-e70079-s002.zip › 7.GSEA analysis/4.MYRF/KEGG_ACUTE_MYELOID_LEUKEMIA.html]

Details for gene set KEGG\_ACUTE\_MYELOID\_LEUKEMIA[GSEA]

|  || Dataset | MYRF.group.cls#h\_versus\_l |
| Phenotype | group.cls#h\_versus\_l |
| Upregulated in class | l |
| GeneSet | KEGG\_ACUTE\_MYELOID\_LEUKEMIA |
| Enrichment Score (ES) | -0.36728093 |
| Normalized Enrichment Score (NES) | -1.5668428 |
| Nominal p-value | 0.05202312 |
| FDR q-value | 0.19884887 |
| FWER p-Value | 0.665 |
Table: GSEA Results Summary

  

Fig 1: Enrichment plot: KEGG\_ACUTE\_MYELOID\_LEUKEMIA      
 Profile of the Running ES Score & Positions of GeneSet Members on the Rank Ordered List

  

| SYMBOL | TITLE | RANK IN GENE LIST | RANK METRIC SCORE | RUNNING ES | CORE ENRICHMENT || 1 | PIK3R5 | na | 661 | 0.528 | 0.0467 | No |
| 2 | FLT3 | na | 5327 | 0.212 | -0.0614 | No |
| 3 | SPI1 | na | 5859 | 0.197 | -0.0522 | No |
| 4 | SOS1 | na | 6706 | 0.172 | -0.0551 | No |
| 5 | PIK3R1 | na | 7105 | 0.161 | -0.0465 | No |
| 6 | BRAF | na | 8198 | 0.133 | -0.0614 | No |
| 7 | MTOR | na | 8431 | 0.129 | -0.0521 | No |
| 8 | CCNA1 | na | 9171 | 0.112 | -0.0594 | No |
| 9 | KRAS | na | 9371 | 0.108 | -0.0517 | No |
| 10 | PIK3CG | na | 9794 | 0.100 | -0.0515 | No |
| 11 | AKT2 | na | 10210 | 0.092 | -0.0520 | No |
| 12 | CEBPA | na | 10512 | 0.086 | -0.0499 | No |
| 13 | PIK3CA | na | 11255 | 0.074 | -0.0621 | No |
| 14 | RPS6KB2 | na | 11604 | 0.069 | -0.0635 | No |
| 15 | PIK3R2 | na | 12535 | 0.056 | -0.0834 | No |
| 16 | SOS2 | na | 12783 | 0.053 | -0.0839 | No |
| 17 | IKBKB | na | 12994 | 0.050 | -0.0837 | No |
| 18 | RPS6KB1 | na | 13890 | 0.039 | -0.1046 | No |
| 19 | MAP2K2 | na | 14012 | 0.038 | -0.1034 | No |
| 20 | CHUK | na | 14348 | 0.034 | -0.1089 | No |
| 21 | MAPK3 | na | 14382 | 0.034 | -0.1056 | No |
| 22 | CCND1 | na | 14657 | 0.031 | -0.1097 | No |
| 23 | TCF7L2 | na | 15138 | 0.027 | -0.1202 | No |
| 24 | STAT5B | na | 15287 | 0.025 | -0.1214 | No |
| 25 | PIK3CB | na | 17199 | 0.007 | -0.1756 | No |
| 26 | AKT1 | na | 18101 | -0.001 | -0.2014 | No |
| 27 | PPARD | na | 18532 | -0.006 | -0.2131 | No |
| 28 | JUP | na | 19857 | -0.020 | -0.2488 | No |
| 29 | MAPK1 | na | 20406 | -0.027 | -0.2613 | No |
| 30 | PIK3CD | na | 20433 | -0.027 | -0.2587 | No |
| 31 | PIK3R3 | na | 20464 | -0.027 | -0.2562 | No |
| 32 | KIT | na | 21671 | -0.042 | -0.2858 | No |
| 33 | IKBKG | na | 21687 | -0.042 | -0.2810 | No |
| 34 | RAF1 | na | 21720 | -0.042 | -0.2767 | No |
| 35 | HRAS | na | 21856 | -0.044 | -0.2751 | No |
| 36 | BAD | na | 23795 | -0.072 | -0.3220 | No |
| 37 | RARA | na | 23932 | -0.074 | -0.3167 | No |
| 38 | GRB2 | na | 24215 | -0.079 | -0.3149 | No |
| 39 | PML | na | 25100 | -0.095 | -0.3286 | No |
| 40 | STAT5A | na | 25954 | -0.112 | -0.3393 | No |
| 41 | STAT3 | na | 26744 | -0.129 | -0.3460 | No |
| 42 | EIF4EBP1 | na | 27483 | -0.147 | -0.3489 | Yes |
| 43 | TCF7L1 | na | 27666 | -0.152 | -0.3353 | Yes |
| 44 | MYC | na | 27707 | -0.153 | -0.3174 | Yes |
| 45 | NFKB1 | na | 28241 | -0.168 | -0.3119 | Yes |
| 46 | ARAF | na | 28444 | -0.174 | -0.2961 | Yes |
| 47 | NRAS | na | 30019 | -0.224 | -0.3136 | Yes |
| 48 | TCF7 | na | 30107 | -0.227 | -0.2878 | Yes |
| 49 | MAP2K1 | na | 30150 | -0.229 | -0.2605 | Yes |
| 50 | PIM2 | na | 30730 | -0.251 | -0.2459 | Yes |
| 51 | RELA | na | 31135 | -0.271 | -0.2239 | Yes |
| 52 | ZBTB16 | na | 31649 | -0.298 | -0.2015 | Yes |
| 53 | PIM1 | na | 31902 | -0.312 | -0.1700 | Yes |
| 54 | RUNX1 | na | 32001 | -0.317 | -0.1333 | Yes |
| 55 | RUNX1T1 | na | 32331 | -0.338 | -0.1007 | Yes |
| 56 | LEF1 | na | 33408 | -0.432 | -0.0780 | Yes |
| 57 | AKT3 | na | 34696 | -0.932 | 0.0009 | Yes |
Table: GSEA details [plain text format]

  

Fig 2: KEGG\_ACUTE\_MYELOID\_LEUKEMIA      
 Blue-Pink O' Gram in the Space of the Analyzed GeneSet

  

Fig 3: KEGG\_ACUTE\_MYELOID\_LEUKEMIA: Random ES distribution      
 Gene set null distribution of ES for **KEGG\_ACUTE\_MYELOID\_LEUKEMIA**
